# The bisphosphonates alendronate and zoledronate induce adaptations of aerobic metabolism in permanent human endothelial cells

**DOI:** 10.1038/s41598-023-43377-3

**Published:** 2023-09-27

**Authors:** Adrianna Budzinska, Lukasz Galganski, Wieslawa Jarmuszkiewicz

**Affiliations:** Laboratory of Mitochondrial Biochemistry, Department of Bioenergetics, Adam Mickiewicz University, Collegium Biologicum, Uniwersytetu Poznanskiego 6, 61-614 Poznan, Poland

**Keywords:** Biochemistry, Cell biology

## Abstract

Nitrogen-containing bisphosphonates (NBPs), compounds that are widely used in the treatment of bone disorders, may cause side effects related to endothelial dysfunction. The aim of our study was to investigate the effects of chronic 6-day exposure to two common bone-preserving drugs, alendronate and zoledronate, on endothelial function and oxidative metabolism of cultured human endothelial cells (EA.hy926). NBPs reduced cell viability, induced oxidative stress and a pro-inflammatory state and downregulated the prenylation-dependent ERK1/2 signaling pathway in endothelial cells. In addition, NBPs induced increased anaerobic respiration and slightly increased oxidative mitochondrial capacity, affecting mitochondrial turnover through reduced mitochondrial fission. Moreover, by blocking the mevalonate pathway, NBPs caused a significant decrease in the level of coenzyme Q10, thereby depriving endothelial cells of an important antioxidant and mitochondrial electron carrier. This resulted in increased formation of reactive oxygen species (ROS), upregulation of antioxidant enzymes, and impairment of mitochondrial respiratory function. A general decrease in mitochondrial respiration occurred with stronger reducing fuels (pyruvate and glutamate) in NBP-treated intact endothelial cells, and significantly reduced phosphorylating respiration was observed during the oxidation of succinate and especially malate in NBP-treated permeabilized endothelial cells. The observed changes in oxidative metabolism caused a decrease in ATP levels and an increase in oxygen levels in NBP-treated cells. Thus, NBPs modulate the energy metabolism of endothelial cells, leading to alterations in the cellular energy state, coenzyme Q10 redox balance, mitochondrial respiratory function, and mitochondrial turnover.

## Introduction

Bisphosphonates (BPs), the synthetic analogs of pyrophosphate, are among the most commonly prescribed drugs worldwide due to their effectiveness in the treatment of osteoporosis, other less common bone pathologies, and certain bone cancers^1,2^. BPs inhibit bone resorption by impeding osteoclast activity or by inducing apoptosis^3^. Nitrogen-containing bisphosphonates (NBPs) inhibit farnesyl diphosphate synthase (FPPS), a key enzyme of the intracellular mevalonate pathway, thereby preventing prenylation and activation of the small GTPases that are essential for bone resorption and osteoclast survival. The BP-inhibited mevalonate pathway is also responsible for the biosynthesis of cholesterol, other sterols, isoprenoid lipids, heme *a*, coenzyme Q^4,5^.

Coenzyme Q is a key electron carrier in the mitochondrial respiratory chain and an important antioxidant that is present in all cell membranes^6^. In addition, it is involved in the production of mitochondrial reactive oxygen species (ROS) through the mitochondrial respiratory chain. A decrease in coenzyme Q levels, resulting from the blockage of the mevalonate pathway, may result in abnormal mitochondrial respiratory function, leading to oxidative damage^6,7^. Currently, there are few studies of the effects of NBPs on cell coenzyme Q10 levels. For example, NBP therapy has been associated with impaired coenzyme Q10 status in the plasma of postmenopausal women^8^.

Endothelial cells line all blood vessels, and thus they are among the first cells that contact drugs such as BPs that are transported by the blood. Therefore, endothelial oxidative metabolism may be susceptible to changes in blood components, thereby contributing to oxidative stress. Endothelial dysfunction is closely related to the excessive production of ROS, including those in the mitochondria, and therefore may lead to the development of civilization diseases of the cardiovascular system^9–12^.

Although the skeletal system is the primary target of BPs, studies have shown that these anti-osteoporosis drugs also affect the function of endothelial cells; the latter play key roles in vascular metabolism and homeostasis^13^. Prolonged or high doses of NBPs can cause adverse effects, including an increased risk of cardiovascular events or BP-related osteonecrosis of the jaw (BRONJ) in association with inhibition of angiogenesis^14–16^. NBPs, especially zoledronate, have been demonstrated to affect cell viability, cell migration, and apoptosis of human umbilical vein endothelial cells (HUVECs)^17^. In addition, NBPs negatively affect angiogenesis by inhibiting adhesion, proliferation, survival, migration, and formation of actin stress filaments in HUVECs by interfering with protein prenylation^18–21^.

The effect of NBPs on the oxidative metabolism of endothelial cells, a process related to the level of coenzyme Q10, has not yet been characterized. Understanding the mechanisms by which NBPs affect the energy metabolism of endothelial cells is important to elucidate their potential impact on the proper functioning of the cardiovascular system.

The aim of our study was to investigate the effects of chronic 6-day exposure to two common bone-preserving NBPs, alendronate and zoledronate, on the endothelial function and aerobic metabolism of cultured human endothelial EA.hy926 cells. Coenzyme Q10 content, ROS production, ATP level and mitochondrial respiratory function were studied in control and bisphosphonate-treated cells. In addition, we examined the effects of NBPs on cell viability, markers of inflammation and oxygen level, and mitochondrial turnover.

## Results

### Dose-dependent effects of NBPs on cell viability and cellular coenzyme Q10 content

We selected two representative NBPs for the study: zoledronate, a potent bisphosphonate due to its unique R2 side chain consisting of a heterocyclic ring containing two nitrogen atoms, and alendronate, an NBP that has medium-level potency^22^. The micromolar concentrations used in the experiments were selected from a wide range of published studies on the concentrations of these compounds found in the serum of patients treated for osteoporosis or bone cancer after drug infusions^23–25^.

**Figure 1:**
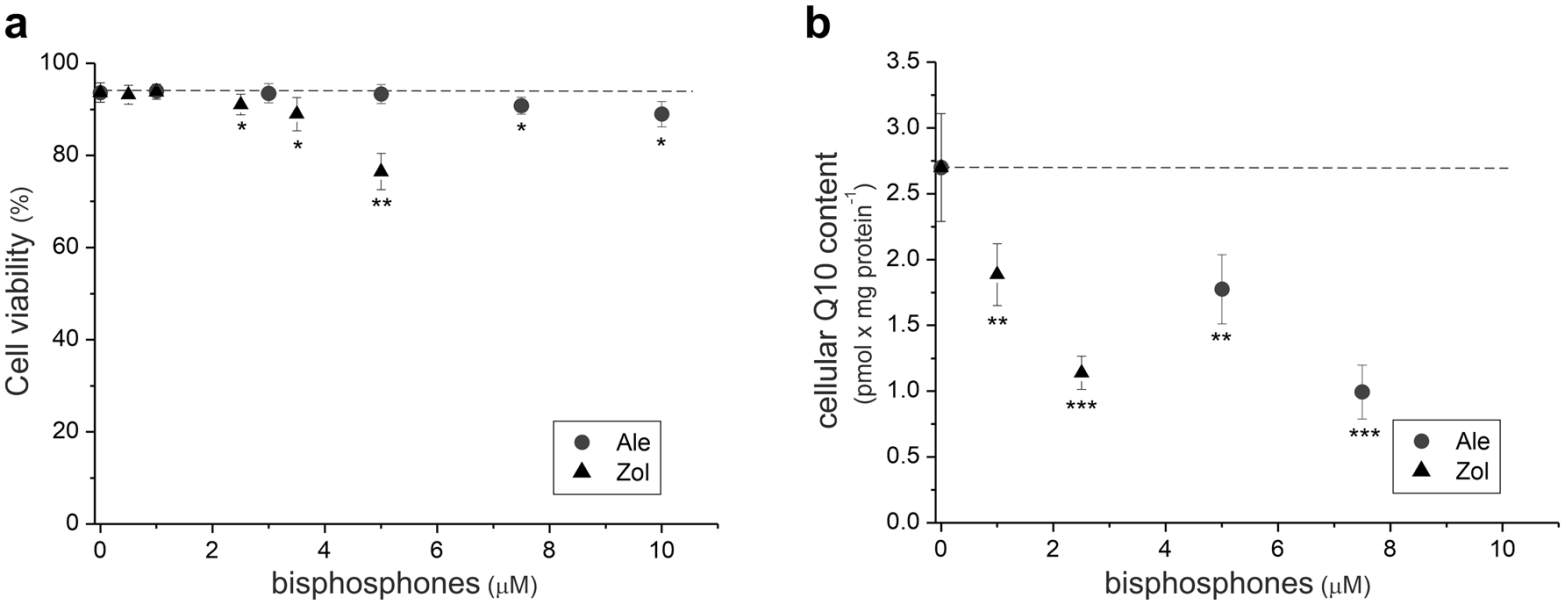
Dose-dependent effects of NBPs on viability (**a**) and cellular Q10 content (**b**) of endothelial cells (EA.hy926). Mean ± SD; *n* = 5. *P* < 0.05 (*), *P* < 0.01 (**), *P* < 0.001 (***), comparison *vs*. control cells (Ctr). Ale, alendronate; Zol, zoledronate.

Our initial goal was to select doses of alendronate and zoledronate that significantly affected the cellular Q10 content without impacting endothelial cell viability. We studied the effects of NBPs on endothelial cell viability using concentrations of 1–10 µM for alendronate and 0.5–5 µM for zoledronate (Fig. 1a). Higher concentrations (from 7.5 µM for alendronate and 2.5 µM for zoledronate) significantly reduced the viability of endothelial cells (Fig. 1a) and the content of Q10 (by ~ 60% for 7.5 µM alendronate and 2.5 µM zoledronate) in these cells (Fig. 1b). Therefore, subsequent experiments were performed with endothelial cells cultured for six days under controlled conditions without NBPs and with 5 μM alendronate or 1 μM zoledronate, concentrations that did not affect cell viability but significantly (by ~ 30%) reduced total cellular Q10 content.

Our results revealed that chronic exposure of endothelial cells to high concentrations of NBPs decreased cell viability, indicating the cellular toxicity of these compounds. In addition, we have demonstrated for the first time that NBPs significantly reduce cellular Q10 levels and that a severe deficiency of Q10 (~ 60%) may contribute to the loss of cell viability.

### NBP-induced decrease in Q10 levels led to increased ROS formation and upregulation of antioxidant enzymes

**Figure 2:**
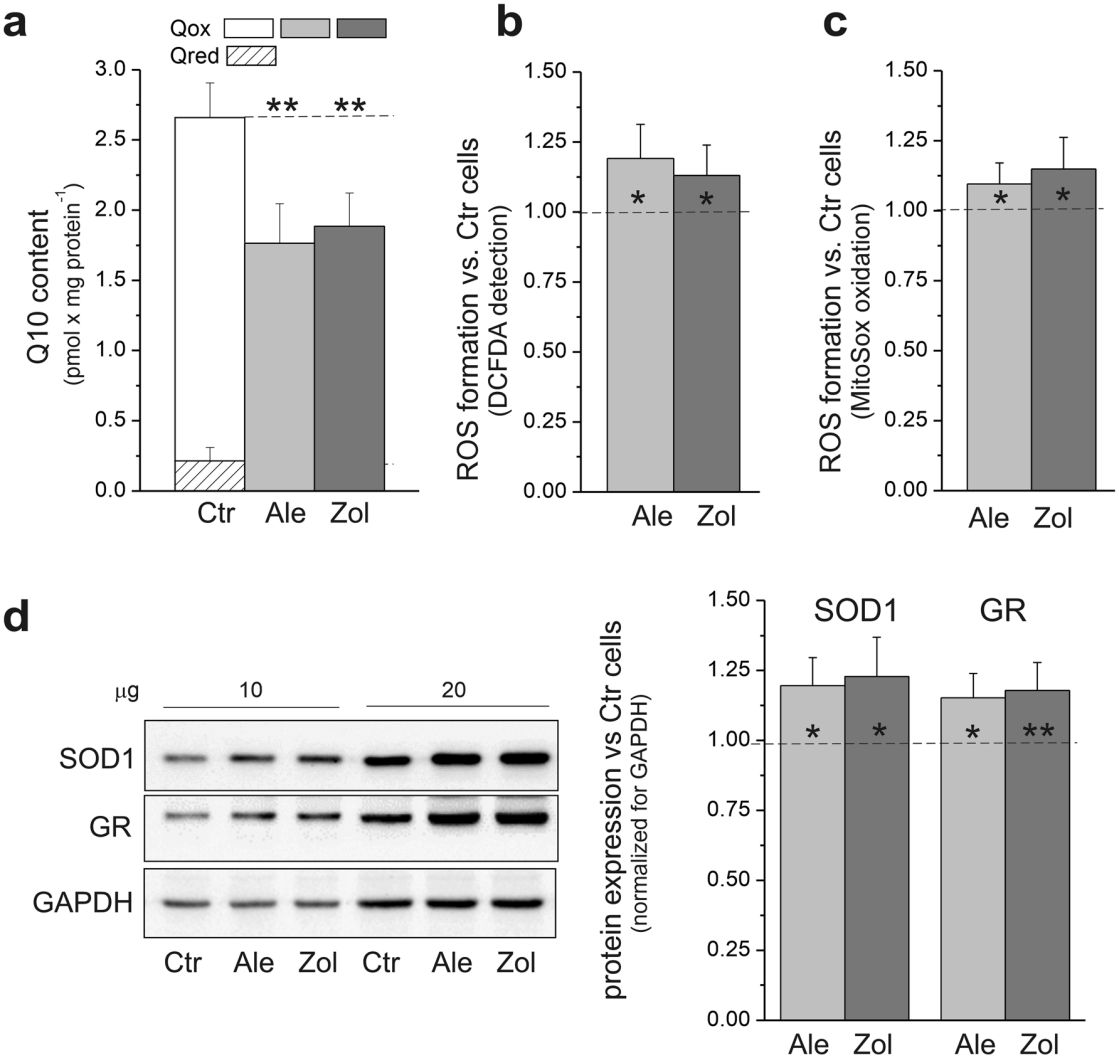
Effect of 5 µM alendronate (Ale) and 1 µM zoledronate (Zol) on total, oxidized, and reduced Q10 pool (**a**), total (**b**) and mitochondrial (**c**) ROS production and expression of antioxidant enzymes (**d**) in endothelial cells. **d**, Representative western blots (cropped blots) and analysis of protein expression are presented. Mean ± SD; *n* = 8. *P* < 0.05 (*), *P* < 0.01 (**), comparison *vs*. control cells (Ctr). SOD1, superoxide dismutase; GR, glutathione reductase.

The ~ 30% NBP-induced decrease in total cellular Q10 content was accompanied by elimination of the reduced Q10 (Q10H_2_) pool that accounted for ~ 8% of the total Q10 (Q10 plus Q10H_2_) pool in untreated cells (Fig. 2a). In endothelial cells treated with NBPs, a decrease in Q10, especially its reduced pool that functions as an antioxidant, led to increases in total cellular and mitochondrial ROS production (Fig. 2b and c). The additional ROS resulted in oxidative stress, as indicated by a 15–20% increase in the expression of the antioxidant enzymes glutathione reductase and superoxide dismutase 1 (Fig. 2d). The effects of 5 µM alendronate and 1 µM zoledronate were similar.

Our results indicated that in endothelial cells treated with NBPs, the greatly decreased Q10 level that led to oxidative stress was compensated for by increases in the other antioxidants (i.e., glutathione reductase and superoxide dismutase 1).

### In endothelial cells, zoledronate significantly increased inflammation markers, while both tested NBPs increased oxygen level marker

**Figure 3:**
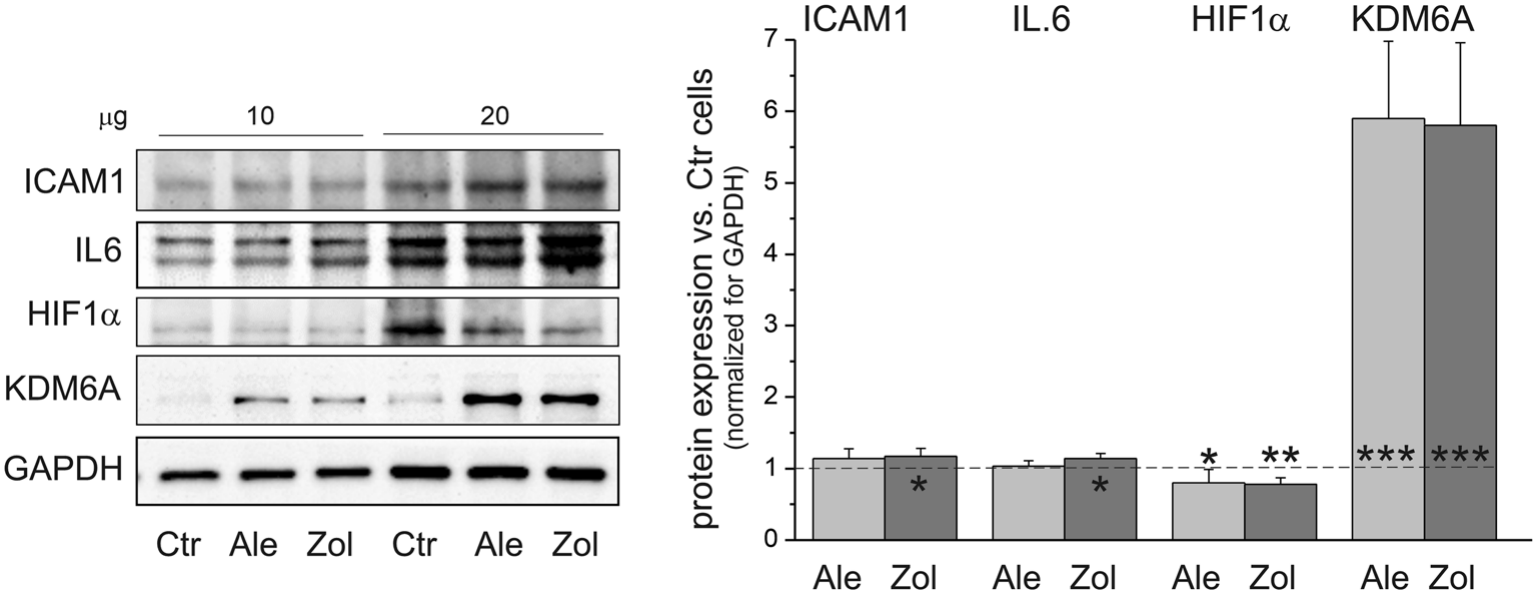
Effect of 6-day endothelial cell culture with 5 µM alendronate (Ale) and 1 µM zoledronate (Zol) on inflammation and oxygen level markers. Representative western blots (cropped blots) and analysis of protein expression are presented. Mean ± SD; *n* = 6. *P* < 0.05 (*), *P* < 0.01 (**), *P* < 0.001 (***), comparison *vs*. control cells (Ctr). ICAM1, intercellular adhesion molecule 1; IL6, interleukin-6; HIF1α, hypoxia-inducible factor 1α; H KDM6A, lysine (K)-specific demethylase 6A.

Intercellular adhesion molecule 1 (ICAM1) is an adhesion receptor that regulates the recruitment of leukocytes from the circulation to endothelial cells at sites of inflammation^26,27^. The cytokine interleukin-6 (IL6) plays a key role in inflammation and directly affects vascular endothelial cells, which produce several types of cytokines and chemokines and activate the coagulation cascade^28^. In our study, increases in the expression of these inflammatory marker proteins were observed in cells treated with zoledronate, but not in alendronate-treated or untreated cells (Fig. 3). However, the applied concentrations of the NBPs (5 µM alendronate and 1 µM zoledronate) had no significant effect on the viability of endothelial cells (Fig. 1a).

NBP-treated cells showed significant (~ 20%) reductions in hypoxia-inducible factor 1α (HIF1α), which is a marker of hypoxia, and a six-fold increase in lysine (K)-specific histone demethylase 6A (KDM6A) that functions as a direct cellular oxygen sensor that regulates gene transcription. Oxygen levels via HIF1α regulate KDM6A to control chromatin and cell fate^29,30^. Our results indicate elevated oxygen levels in NBP-treated cells, which may contribute to oxidative stress and changes in cellular oxidative metabolism.

### In endothelial cells, NBPs upregulated mitochondrial oxidative capacities and anaerobic respiration

**Figure 4:**
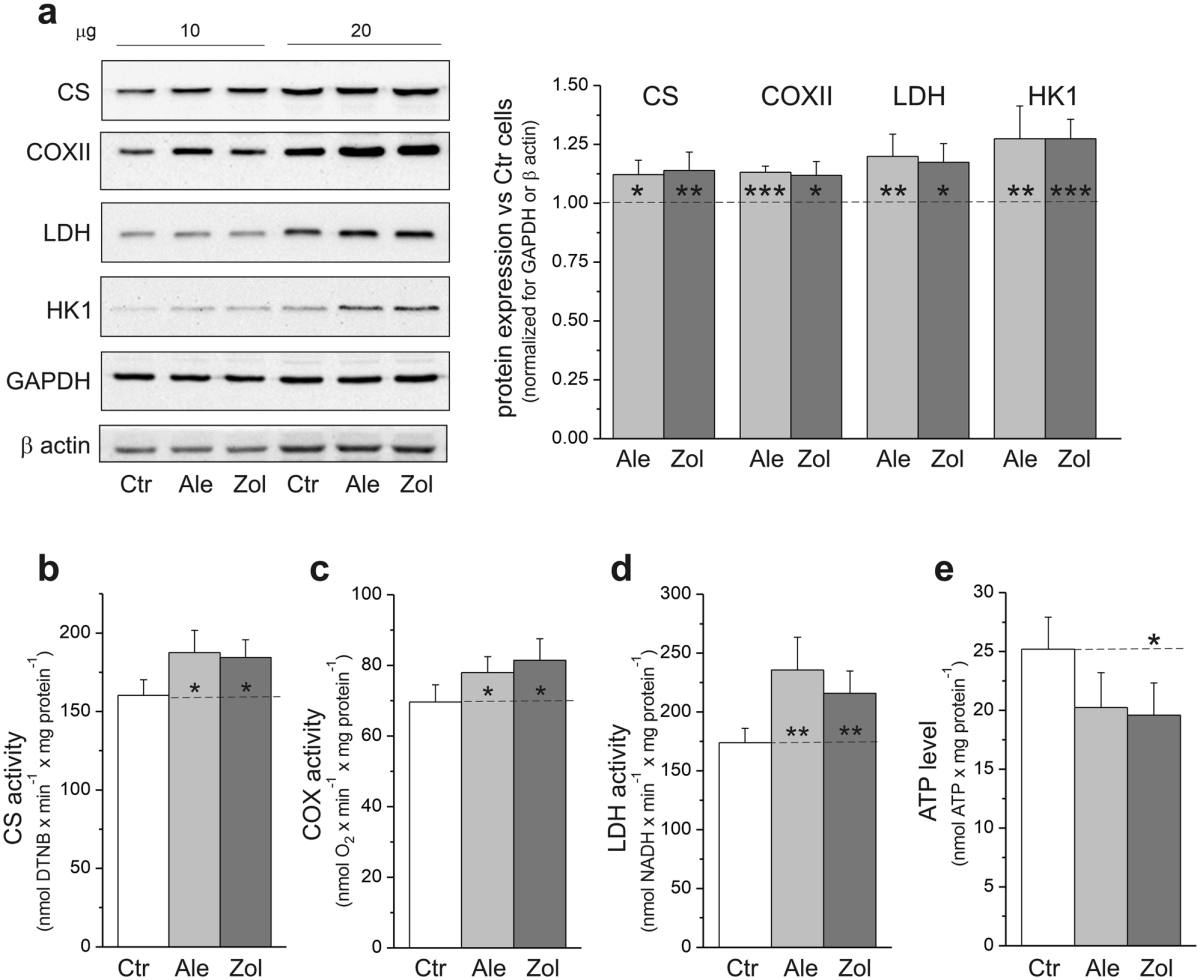
Effect of 6-day endothelial cell culture with 5 µM alendronate (Ale) and 1 µM zoledronate (Zol) on markers of mitochondrial and anaerobic respiration. Representative western blots (cropped blots) and analysis of protein expression (**a**). Protein expression levels were normalized for β actin (lactate dehydrogenase, LDH) or GAPDH (other proteins). Maximal aerobic (**b**, **c**) and anaerobic respiration (**d**) marker activities and ATP level (**e**). Mean ± SD; *n* = 6–7. *P* < 0.05 (*), *P* < 0.01 (**), *P* < 0.001 (***), comparison vs. control cells (Ctr). CS, citric synthase; COXII, cytochrome *c* oxidase; HK1, hexokinase I.

Citrate synthase (CS), the first enzyme of the tricarboxylic acid cycle (TCA cycle), and cytochrome *c* oxidase (COX), complex IV of the respiratory chain, are markers of mitochondrial oxidative function. Compared to control cells, endothelial cells treated with NBPs showed slight (~ 12–17%) increases in the expression levels (Fig. 4a) and activities (Fig. 4b and c) of CS and COX, indicating greater oxidative capacities of the TCA cycle and respiratory chain. The increased mitochondrial oxidative capacities were accompanied by a 27% increase in the expression level of hexokinase-1 (HK1), a key enzyme of glycolysis (Fig. 4a), and a significantly greater expression level (~ 16–20%) and activity (~ 25–35%) of lactate dehydrogenase (LDH) (Fig. 4a and d), an enzyme that converts pyruvate to lactate to maintain increased flux through glycolysis.

Thus, our results indicate that in endothelial cells, NBPs increase the oxidative capacity of mitochondria as well as anaerobic respiration. Despite the increase in respiratory capacity, a decrease (statistically significant in zoledronate-treated cells) in ATP levels was observed in the NBP-treated cells compared to untreated cells (Fig. 4e).

### NBPs affected mitochondrial turnover by reducing mitochondrial fission in endothelial cells

**Figure 5:**
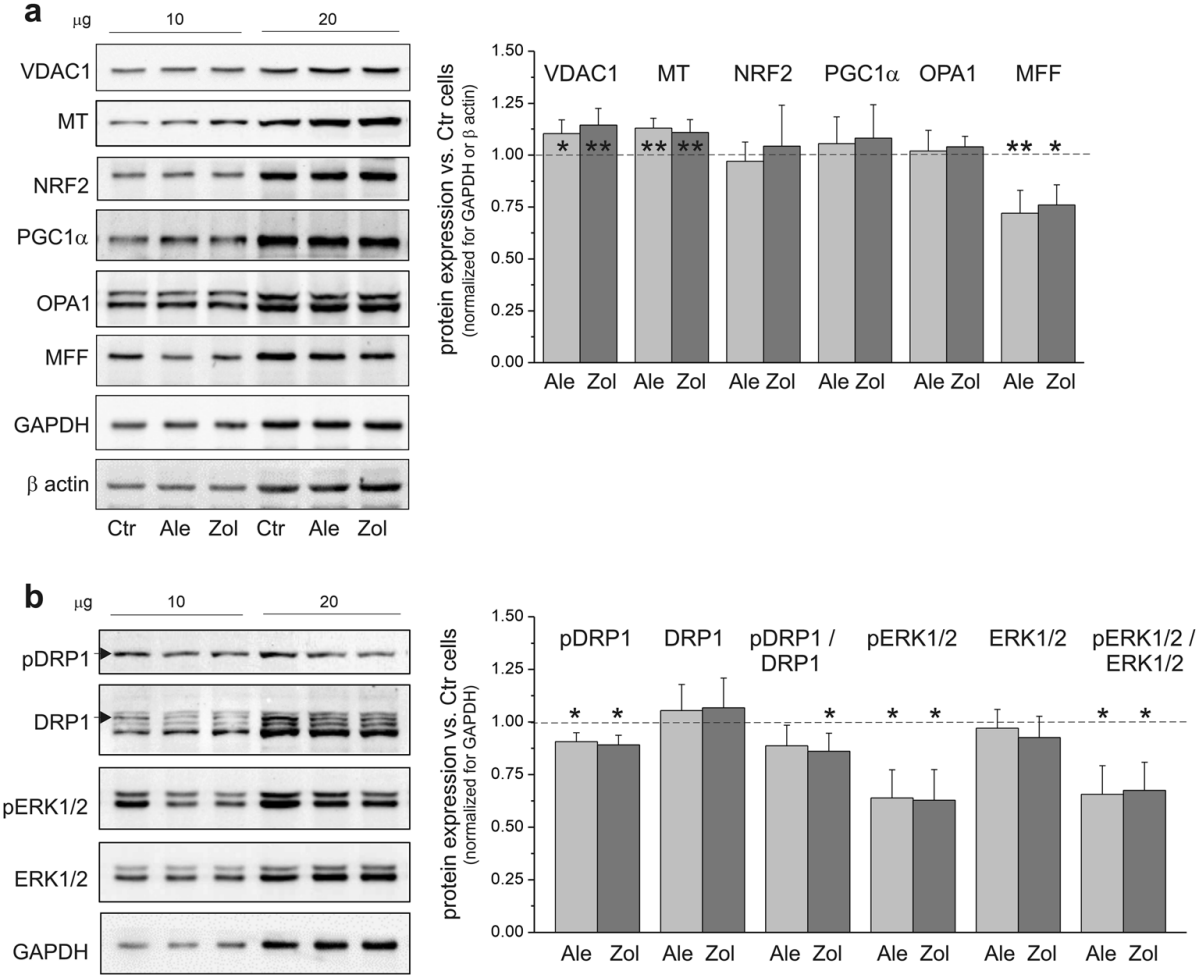
Markers of mitochondrial dynamics of endothelial cells grown with 5 µM alendronate (Ale) and 1 µM zoledronate (Zol). Representative western blots (cropped blots) and analysis of protein expression are presented. Mean ± SD; *n* = 6. *P* < 0.05 (*), *P* < 0.01 (**), comparison *vs*. control cells (Ctr). (**a**) VDAC1, voltage-dependent anion-selective channel protein 1; MT, mitochondrial marker, mitochondrial non-glycosylated protein; NRF2, nuclear factor erythroid 2-related factor, PGC1α, peroxisome proliferator-activated receptor γ coactivator 1α; OPA1, artrophy-1 protein; MFF, mitochondrial cleavage factor. (**b**) pDRP1, phospho-dynamin related protein 1; DRP1, total DRP1 (pDRP1 marked with an arrow); pERK1/2, phospho-extracellular signal-regulated protein kinase 1/2; ERK1/2, total ERK1/2. Protein expression levels were normalized for β actin (MFF) or GAPDH (other proteins).

The greater (up to 17%) levels of mitochondrial marker (CS and COX) activity and expression (Fig. 4) indicates an increased content of mitochondria in endothelial cells treated with NBPs. For example, in human skeletal muscle, CS activity in particular has shown a strong association with mitochondrial content^31^. In our study, increased mitochondrial content in NBP-treated endothelial cells was confirmed by a small (~ 10–15%) increase in the expression of the voltage-dependent anion-selective channel protein 1 (VDAC1) and mitochondrial non-glycosylated protein (MT), another mitochondrial markers (Fig. 5a). Therefore, we have checked NBP-induced changes in mitochondrial turnover markers, including those related to mitochondrial biogenesis and fission/fusion.

Despite the increase in oxidative stress in NBP-treated endothelial cells (increased ROS production) (Fig. 2b and c), no increases in the expression of mitochondrial biogenesis markers, i.e., peroxisome proliferator-activated receptor γ coactivator (PGC1α) and nuclear factor erythroid 2-related factor (NRF2), were observed (Fig. 5a). However, statistically significant decreases in the expression of fission markers, i.e., mitochondrial cleavage factor, MFF and active phospho-dynamin related protein 1, phospho-DRP1 (Ser616) (~ 25% and ~ 12%, respectively), combined with stable levels of a fusion marker (arthrosis protein-1, OPA1) indicated an NBP-induced change in mitochondrial turnover (Fig. 5). Since extracellular signal regulated protein kinase 1/2 (ERK1/2) has previously been shown to prevent inflammatory signaling^32^ as well as regulate DRP1-dependent mitochondrial fusion^33^ in endothelial cells, we also examined the expression levels of active phospho-ERK1/2 (Thr202/Tyr204) in NBP-treated endothelial cells. Figure 5b shows that significantly reduced levels (~ 36%) of phospho-ERK1/2 were observed in endothelial cells treated with NBPs compared to control cells, indicating downregulation of the ERK1/2 signaling pathway.

Our results revealed (i) slightly increased mitochondrial content, (ii) unaltered PGC1α/NRF2 mitochondrial biogenesis signaling pathway, (iii) reduced mitochondrial fission markers accompanied by unchanged fusion marker, and (iv) decreased ERK1/2 signaling pathway favoring the maintenance of the mitochondrial pool when there is a strong decrease in the level of Q10, which, in addition to antioxidant functions, is an important electron carrier in the mitochondrial respiratory chain.

### In endothelial cells, NBPs induced an overall decrease in mitochondrial respiration except for the weak substrates glucose and palmitate

**Figure 6:**
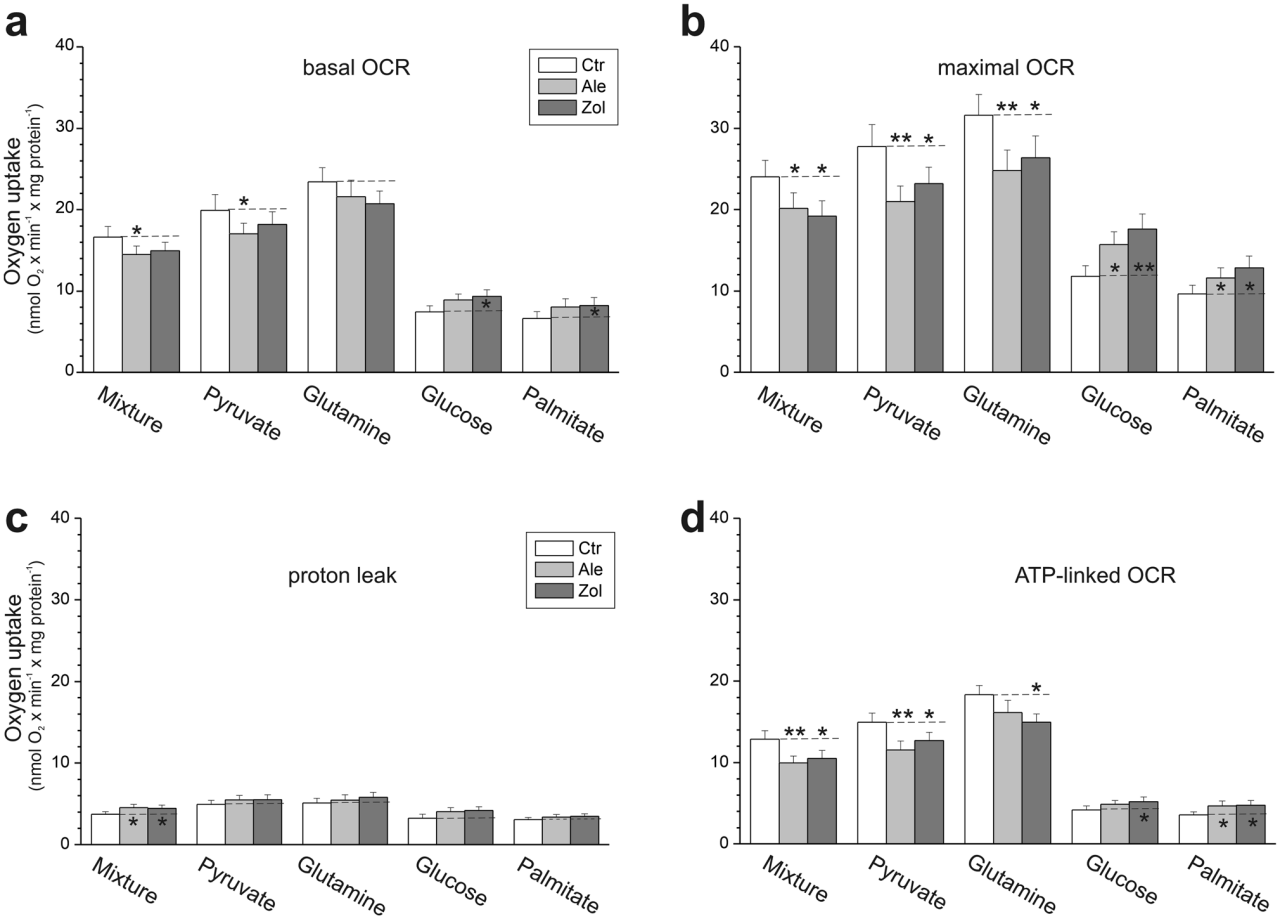
Effect of endothelial cell culture with 5 µM alendronate (Ale) and 1 µM zoledronate (Zol) on mitochondrial oxidative metabolism. Changes in the basal oxygen consumption rate (OCR) (**a**), maximal OCR (**b**), proton leak (**c**), and ATP-linked OCR (**d**) using different reducing substrates. Mean ± SD; *n* = 6. *P* < 0.05 (*), *P* < 0.01 (**), comparison *vs*. control cells (Ctr).

We next analyzed changes in the aerobic metabolism of endothelial cells cultured with 1 µM zoledronate or 5 µM alendronate by measuring the oxidation of different reducing substrates. NBP-treated cells showed increased respiration under uncoupling conditions (maximal oxygen consumption rate, OCR) with comparatively weaker substrates (glucose and palmitate) (Fig. 6). During the oxidation of these substrates, basal OCR and ATP-linked OCR showed statistically significant increases over control cells only in zoledronate treated cells.

However, for more potent respiratory substrates (glutamine, pyruvate and a mixture of all substrates tested), NBP-exposed cells displayed decreased respiration, indicating impairment of mitochondrial respiratory function. For these substrates, NBP-treated cells showed ~ 20–35% reductions in maximal mitochondrial respiratory capacity (maximal OCR) (Fig. 6b) as well as ~ 15–20% decreases in ATP-linked OCR (statistically insignificant for glutamine in alendronate-treated cells) (Fig. 6d), indicating reduced mitochondrial oxidative phosphorylation. In addition, during the oxidation of the mixture of all substrates, an increase in non-ATP-linked OCR (proton leak) was observed (Fig. 6c).

Thus, in NBP-treated cells, we observed (i) a decrease in mitochondrial respiration when strong respiratory substrates pyruvate and glutamine were oxidized alone or mixed, (ii) an increase in proton leak during the oxidation of a mixture of all substrates, and (iii) a decrease in ATP-related respiration with stronger substrates, indicating significant changes in energy metabolism.

### Permeabilized NBP-treated endothelial cells showed decreased phosphorylating respiration and respiratory control ratios in the presence of oligomycin. The effect was more pronounced during complex I substrate oxidation compared to complex II substrate oxidation

Measurement of OCR in permeabilized cells allows for the respiratory activity of mitochondria with complex II (succinate) and complex I (malate) substrates to be determined. In our study, succinate oxidation (complex II activity) was measured in the presence of rotenone to inhibit complex I activity. Permeabilized endothelial cells treated with NBPs showed reduced phosphorylating respiration with both succinate (by 11–15%) and malate (by 22–30%) (Table 1). A reduction in nonphosphorylating respiration was also observed (statistically significant for succinate). Interestingly, in permeabilized NBP-treated endothelial cells during both succinate and malate oxidation, nonphosphorylating respiration was higher (by 11–20%) in the presence of oligomycin, which inhibits ATP conversion. This result indicated increased mitochondrial respiration that was unrelated to ATP synthesis in cells cultured with NBPs, similar to such respiration measured with intact cells (Fig. 6c). However, the level of mitochondrial uncoupling protein 2 (UCP2) was unchanged in NBP-treated cells compared to control cells (Supplementary Fig. S1). In permeabilized NBP-treated endothelial cells, the respiratory control ratio in the absence of oligomycin (RCR) was significantly reduced with malate, while that in the presence of oligomycin (RCR_Oligo_) was decreased for both substrates. RCR_Oligo_ represents the maximal factorial increase in mitochondrial OCR induced by the phosphorylation of ADP to ATP that can be achieved above the proton leak driven OCR when ADP recycling is blocked by oligomycin.

**Table 1:** Respiratory rates and respiratory control ratios in permeabilized control (Ctr), alendronate-treated (Ale), and zoledronate-treated (Zol) endothelial cells.

	Complex I OCR (Malate)					Complex II OCR (Succinate + Rotenone)				
	State 3	State 4	+ Oligo	RCR	RCR_Oligo_	State 3	State 4	+ Oligo	RCR	RCR_Oligo_
	(nmol O × /min × mg/protein)					(nmol O × /min × mg/protein)				
Ctr	8.11 ± 0.70	4.21 ± 0.35	0.89 ± 0.05	1.92 ± 0.09	9.19 ± 0.78	3.96 ± 0.25	3.55 ± 0.21	1.58 ± 0.11	1.12 ± 0.09	2.51 ± 0.18
Ale	5.70 ± 0.37**	3.87 ± 0.31	1.02 ± 0.01*	1.47 ± 0.10*	5.60 ± 0.38***	3.40 ± 0.27*	2.90 ± 0.21*	1.75 ± 0.11*	1.18 ± 0.13	1.94 ± 0.13**
Zol	6.32 ± 0.51*	4.08 ± 0.34	1.02 ± 0.01*	1.55 ± 0.09*	6.21 ± 0.14***	3.52 ± 0.30*	2.98 ± 0.25*	1.91 ± 0.16*	1.18 ± 0.16	1.85 ± 0.14**

State 4, nonphosphorylating respiration; State 3, phosphorylating respiration in the presence of ADP; + Oligo, nonphosphorylating respiration in the presence of oligomycin; RCR, respiratory control ratio (State 3/State 4); RCRoligo, State 3 *vs.* State 4 in the presence of oligomycin (State 3/ + Oligo). Respiratory substrates: succinate (plus 2 μM rotenone) or malate. Mean ± SD; *n* = 5. *P* < 0.05 (*), *P* < 0.01 (**), *P* < 0.001 (***), comparison *vs*. control cells (Ctr).

Thus, our results indicated that endothelial cells cultured with NBPs had (i) greater mitochondrial uncoupling and thus reduced oxidative phosphorylation efficiency, and (ii) respiratory chain inhibition, especially during oxidation of the complex I substrate.

## Discussion

The influence of NBPs on the oxidative metabolism of endothelial cells has yet to be determined. Our results showed that chronic 6-day exposure of endothelial cells (EA.hy926) to either 5 μM alendronate or a five-fold lower concentration of zoledronate caused similar metabolic changes that were not statistically different between the two NBPs.

Exposure of endothelial cells to higher concentrations of NBPs (above 7.5 μM for alendronate and above 2.5 μM for zoledronate) resulted in a decrease in cell viability, indicating the cellular toxicity of these compounds (Fig. 1). NBPs such as zoledronate and alendronate negatively affected angiogenesis in HUVECs by inhibiting cell adhesion, proliferation, viability, and migration^17–21^. Thus, our study supports previous research showing that higher concentrations of NBPs can cause dysfunction of the endothelium, and this may contribute to various side effects of these anti-osteoporosis drugs due to the importance of this tissue in vascular homeostasis. In particular, long-term use or high doses of NBPs can result in adverse reactions such as osteonecrosis of the jaw, musculoskeletal pain, and atypical fractures of long bones^1,8,14,15,21^.

Impaired endothelial function is characterized by increased oxidative stress and a pro-inflammatory state. Zoledronate has been shown to impede endothelial cell function and survival by inhibiting multiple prenylation-dependent signaling pathways, including ERK1/2^18^. Importantly, ERK1/2 also serves as an anti-inflammatory signal to prevent inflammatory signaling in endothelial cells^32^. The endothelial cells treated with zoledronate in our study showed a significant decrease in the level of active phospho-ERK1/2 protein (Fig. 5b) accompanied by increases in the levels of the IL6 and ICAM1 inflammatory markers (Fig. 3). These results indicate that the activation of zoledronate-treated endothelial cells triggered local inflammation by inducing the expression of inflammatory cytokines and adhesion molecules. As in our experiments, previous studies have shown that in osteoblasts and fibroblasts zoledronate, unlike alendronate (even at a five-fold lower concentration), induces an increased level of inflammatory cytokines^34^. In rat endothelial cells, 5 µM alendroante also does not elevate ICAM1^35^. Thus, these observations indicate that zoledronate induces a stronger inflammatory response compared to alendronate, although the effect of both NBPs on ERK 1/2 phosphorylation was similar.

No studies concerning the effects of NBPs on cell coenzyme Q10 levels have been reported, although NBP therapy has been associated with impaired coenzyme Q10 status in the plasma of postmenopausal women^8^. This study is the first to demonstrate that 5 μM alendronate and 1 μM zoledronate significantly reduced cellular Q10 levels, thereby depriving endothelial cells of an important antioxidant and mitochondrial electron carrier, a result that could contribute to a loss of cell viability (Figs. 1 and 2a). The ~ 30% NBP-induced decrease in total cellular Q10 content, accompanied by elimination of the reduced Q10H_2_ pool (Fig. 2a) that functions as an antioxidant, resulted in increases in total and mitochondrial ROS formation (Fig. 2b and c). Thus, NBPs disturbed the cellular Q10 redox homeostasis, thereby decreasing the Q10 redox state (Q10H_2_/Q10) from 0.09 to 0. In endothelial cells treated with NBPs, the Q10 deficiency was only partially restored by other antioxidants (i.e., glutathione reductase and superoxide dismutase 1), leading to oxidative stress (Fig. 2d). Thus, NBPs were able to induce a Q10 deficiency and oxidative stress in endothelial cells by inhibiting FPPS, a key enzyme of the intracellular mevalonate pathway. Previously, significant reductions in Q10 levels resulting in oxidative stress have been observed in endothelial cells treated with the statin atorvastatin, an inhibitor of 3-hydroxy-3-methylglutaryl coenzyme A (HMG-CoA) reductase, another important enzyme in the mevalonate pathway^7^.

Our results showed that NBPs increased mitochondrial oxidative capacity and anaerobic respiration in endothelial cells in terms of CS/COX and LDH activities and expression levels, respectively. However, although there was an increase in the respiratory capacity, a decrease in ATP levels was observed in the NBP-treated cells, indicating an impairment of energy metabolism (Fig. 4e). Despite the slight increase in the mitochondrial content (the amounts of the mitochondrial proteins COX, CS, and VDAC1) (Fig. 4a), the levels of the mitochondrial biogenesis markers PGC1α and NRF2 were unchanged in the NBP-exposed cells (Fig. 5a). In addition, these cells showed a reduced level of the mitochondrial fission markers (MFF and phospho-DRP1) but no change in the level of the mitochondrial fusion marker OPA1 (Fig. 5a). Alternating fission and fusion processes help the mitochondrial network adapt to changing cellular metabolic conditions and allow for the mixing and dissemination of mitochondrial metabolites and enzymes^36^. In our study, reduced mitochondrial fission markers in NBP-treated endothelial cells could indicate less mitochondrial clearance by mitophagy resulting from the downregulation of ERK1/2 signaling (Fig. 5b), the pathway that controls mitochondrial fission and intracellular apoptosis^36^. By inhibiting FPPS and thereby preventing prenylation and activation of small GTPases, NBPs could reduce prenylation-dependent ERK1/2 signaling and the related mitochondrial fission machinery; this may influence metabolic reprogramming. Thus, NBP-induced changes in mitochondrial turnover in endothelial cells may result from dysregulated ERK signaling leading to impaired fission despite oxidative stress that supposedly promotes fission to remove damaged part by mitophagy and maintain mitochondrial function. Therefore, when mitochondrial fission is impaired due to NBPs, the mitochondrial pool may be preserved even with Q10 deficiency. However, further studies should evaluate changes in mitochondrial turnover induced by NBPs by examining mitochondrial morphology.

An overall decrease in mitochondrial respiration was observed in NBP-treated endothelial cells when stronger reducing substrates were used (pyruvate, glutamine, and a mixture of all tested substrates) (Fig. 6b), indicating a reduction in the upper limit of oxygen consumption that was possibly related to coenzyme Q10 deficiency (Fig. 2a), despite the greater total cell respiratory capacity (expressed by COX activity, Fig. 4c). With the weaker reducing substrates (glucose and palmitate) that did not reach this limit (Fig. 6b), the increase in oxidation observed was likely related to the higher mitochondrial content in the NBP-treated cells. In addition, the limitation of the mitochondrial respiratory chain function resulting from the coenzyme Q10 deficiency was indicated by significantly reduced phosphorylating respiration during the oxidation of the complex II substrate (succinate) and especially the complex I substrate (malate) in NBP-treated permebilized endothelial cells (Table 1). The increase in proton leakage in NBP-treated intact cells (Fig. 6c) and the increased nonphosphorylating respiration in the presence of oligomycin together with the decreased respiration control ratio in permeabilized NBP-treated cells (Table 1) indicate increased mitochondrial uncoupling, which can reduce the efficiency of oxidative phosphorylation (ATP synthesis). Impairment of the oxidative phosphorylation system was also indicated by reduced ATP-related respiration with stronger reducing substrates in NBP-treated intact cells (Fig. 6d), decreased ATP levels in these cells (Fig. 4e), and significantly lower phosphorylating respiration independent of the respiratory substrate in permeabilized NBP-treated cells (Table 1). These significant changes in the energy metabolism of NBP-treated endothelial cells caused a significant reduction in oxygen consumption (Fig. 6, Table 1) that in turn led to an increase in the oxygen level in the cells, as indicated by the decrease in the level of HIF1α and a six-fold increase in the expression level of KDM6A, a direct cellular oxygen sensor. NBP-induced increases in the oxygen levels of endothelial cells can contribute to oxidative stress. To our knowledge, this is the first study that links coenzyme Q10 deficiency to increased cellular oxygen levels. Changes in the oxidative metabolism of endothelial cells due to an inhibition of the mevalonate pathway have been reported in a study concerning the statin atorvastatin^7^. Similar to NBP-induced changes (this study), the atorvastatin-induced changes in endothelial cells included coenzyme Q10 deficiency, oxidative stress induction, and decreased mitochondrial respiration^7^. Interestingly, statins did not affect mitochondrial biogenesis in endothelial cells.

In conclusion, our results confirm previous reports that higher concentrations of NBPs may negatively affect angiogenesis by inhibiting endothelial cell viability, thereby causing various side effects associated with these anti-osteoporosis drugs. In addition, our study shows for the first time that NBPs, especially zoledronate, can modulate the energy metabolism of endothelial cells, leading to alterations in the energy state of cells, the coenzyme Q10 redox balance, mitochondrial respiratory function, and mitochondrial turnover. NBPs induced metabolic reprogramming in endothelial cells in response to the deficiency of coenzyme Q10, a cellular antioxidant and a key electron carrier in the mitochondrial respiratory chain.

## Material and methods

### Cell culture and cell fraction preparation

We used the stable human endothelial cell line EA.hy926 (ATCC CRL-2922, ATCC, Manassas, VA, USA) that was originally derived from the human umbilical vein. Endothelial cells were cultured in Dulbecco’s modified Eagle’s medium (DMEM) supplemented with 10% fetal bovine serum (FBS), 1% l-glutamine, 2% hypoxanthine-aminopterin-thymidine (HAT), and 1% penicillin/streptomycin at 5% CO_2_ and 37 °C. Cells were cultured under controlled conditions or in the presence of one of the bisphosphonates alendronate or zoledronate dissolved in water (pH 7.0). Cells from passages 4–12 were grown in 140 mm dishes to approximately 90–100% confluence. Bisphosphonates were added on the day of passage to a final concentration of 5 µM alendronate or 1 µM zoledronate (except for the studies described in Fig. 1).

After 6 days of culture, cells from the control and NBP-treated cultures were harvested with trypsin/ethylenediaminetetraacetic acid (EDTA), washed with 10% FBS in phosphate-buffered saline (PBS), 5% FBS in PBS, and finally with PBS alone, and centrifuged at 1200 × *g* for 10 min at 4 °C. Finally, the cell pellets were resuspended in cold PBS (1 g of cells per 3 ml of PBS) and maintained on ice. The yield of harvested cells was similar in the control and NBP-treated cells, with approximately 1 g of cells per 10 dishes (when all cell types were plated at the same density).

The cells were homogenized 10 times for 5 s in PBS using a polytron (IKA T18, IKA-Werke GmbH & Co. KG, Staufen, Germany) to obtain cytosolic fractions for enzyme measurements. Unbroken cells and cell debris were removed by centrifugation of the homogenates at 1200 × *g* for 10 min at 4 °C. Homogenate supernatants were collected to measure citrate synthase (CS) and lactate dehydrogenase (LDH) activities.

### Enzyme activities in cytosolic fractions

Citrate synthase (CS) activity was determined spectrophotometrically by following the formation of 5,5′-dithiobis(2-nitrobenzoic acid)-Coenzyme A (DTNB-CoA) at 412 nm using a Shimadzu UV 1620 spectrophotometer (Shimadzu Corporation, Kyoto, Japan), as previously described^7^. The reaction mixture contained 100 µM oxaloacetate, 100 μM acetyl-CoA, 100 μM DTNB, 0.1% Triton X-100, 100 mM Tris/HCl (pH 8.0) and 100 µg protein/ml cytosolic fraction.

Lactate dehydrogenase (LDH) activity was measured spectrophotometrically at 340 nm during the oxidation of 200 µM NADH in the presence of 20 mM pyruvate, 50 mM Tris/HCl (pH 7.3) and 100 µg protein/ml cytosolic fraction^37^.

Enzymatic measurements were conducted at 37 °C with constant stirring.

### Cell respiration

Cell oxygen consumption was measured polarographically at 37 °C using a Clark-type oxygen electrode (Hansatech Instruments Ltd, Pentney, UK) in 0.6 ml of DMEJ containing 0.8 mM MgSO_4_, 5.4 mM KCl, 110 mM NaCl, 1.1 mM NaH_2_PO_4_, 44 mM NaHCO_3_ and 10 mM Na/Na buffer (pH 7.5) as previously described^7,38^. The measurement was performed with cells at a final concentration of 4 mg protein/ml. The respiratory substrates used were 5.5 mM glucose, 5 mM pyruvate, 4 mM glutamine, 0.3 mM palmitate, and a mixture of these. To estimate proton leakage, i.e., non-ATP-linked oxygen consumption rate (OCR), ATP synthesis in basal respiration was inhibited with oligomycin (1 μg/ml). The maximal OCR was then determined by adding up to 0.5 µM carbonyl-*p*-trifluoromethoxyphenylhydrazone cyanide (FCCP), as an uncoupler. No residual (non-mitochondrial) respiration was observed in the presence of 0.5 mM cyanide.

The maximal activity of cytochrome *c* oxidase (COX) in cell fractions was measured as the rate of oxygen consumption during oxidation with up to 2 mM N,N,N′N′-tetramethyl-*p*-phenylenediamine (TMPD) in the presence of 10 μM antimycin A and 8 mM ascorbate^39^.

To assess mitochondrial respiration, we measured the oxidation of the complex I (5 mM malate) and complex II (5 mM succinate plus 2 μM rotenone) substrates in endothelial cells permeabilized with 0.02% digitonin. The measurement was performed with cells at a final concentration of 6.7 mg protein/ml in 0.6 ml of incubation medium (at 37 °C) containing 150 mM sucrose, 2 mM MgCl_2_, 2.5 mM KH_2_PO_4_, 20 mM Tris/HCl (pH 7.2) and 0.1% bovine serum albumin (BSA). Phosphorylating (state 3) respiration was measured in the presence of 150 µM ADP. Nonphosphorylating (resting state, state 4) respiration was measured in the absence or presence of oligomycin (1 µg/mg/protein) an inhibitor of ATP synthase. Respiratory control ratio (RCR) was calculated as the state 3 respiratory rate attained during maximal ATP synthesis (i.e., in the presence of ADP) divided by the respiratory rate in the absence of added ADP (in the presence or absence of oligomycin).

### Cell viability

A 0.4% trypan blue solution (1:1 v/v) was added to the harvested live and dead endothelial cells and cell viability was determined using a Countess Automatic Cell Counter (Invitrogen, Carlsbad, CA, USA).

### ATP level detection

ATP levels in endothelial cells were measured using the Luminescent ATP Detection Assay kit (ab113849, Abcam, Cambridge, UK). After cell lysis, luciferase and luciferin were added and the emitted light was detected using a Tecan Spark multiplate reader (Tecan Group Ltd, Mannedorf, Switzerland).

### ROS formation

Total cellular ROS formation was measured with a 5 mM 5-(and-6)-chloromethyl-2′,7′-dichlorodihydrofluorescein diacetate, acetyl ester (CM-H2DCFDA) probe, and mitochondrial ROS (superoxide) formation was determined using a 5 µM MitoSox Red probe. Cells (50 µg protein/ml) were incubated with fluorescent probes in PBS containing 5.5 mM glucose and 5 mM pyruvate for 10 min at 37 °C. The cells were then washed twice with PBS, centrifuged (1200 × *g* for 10 min at 4 °C) and resuspended in PBS to a concentration of 50 μg protein/ml. Measurements were performed in 96-well plates with MitoSox (Excitation/Emission at 510/595 nm) and CM-H2DCFDA (Excitation/Emission at 495/522 nm) using a Tecan Spark multiplate reader (Tecan Group Ltd).

### Cellular Q10 concentration

Cellular Q10 levels were determined by extraction and high-performance liquid chromatography (HPLC), as previously described^40,41^ using a LiChrosorb RP*-*18 (10 µm) HPLC column (Hichrom, Theale, UK). Both the reduced (275 nm) and oxidized (290 nm) forms of Q10 were detected. Commercial Q10 was used to quantify and calibrate the Q10 peaks. Total and reduced Q10 pools were determined in endothelial cells under fully oxidizing conditions, i.e., in the absence of Q-reducing respiratory substrates. Prior to Q extraction, the cells (30 mg) were incubated with gentle agitation for 10 min in 3 ml of PBS.

### Protein immunodetection

Endothelial cells were lysed with RIPA buffer (150 mM NaCl, 0.5% sodium deoxycholate, 1% Triton X-100, 0.1% SDS and 50 mM Tris/HCl, pH 8.0). 8–12% SDS-PAGE gels were used for protein separation. The Spectra™ Multicolor Broad Range Protein Ladder (Thermo Fisher Scientific, Waltham, MA, USA) was used as a molecular weight marker. The following primary antibodies were obtained from Abcam: rabbit polyclonal anti-citrate synthase (CS, 46 kDa) (ab96600), anti-cytochrome *c* subunit II (COXII, 24 kDa) (ab110258), anti-voltage-dependent anion-selective channel protein 1 (VDAC1, 35 kDa) (ab14734), anti-nuclear factor erythroid 2-related factor (NRF2, 68 kDa) (ab137550), anti-peroxisome proliferator-activated receptor γ coactivator 1α (PGC1α, 92 kDa) (ab54481), anti-mitochondrial cleavage factor (MFF, 37 kDa) (ab81127), anti-glutathione reductase (GR, 50 kDa) (ab128933), anti-superoxidase dismutase 1 (SOD1, 18 kDa) (ab13498), anti-intercellular adhesion molecule 1 (ICAM1, 90 kDa) (ab53013), anti-mitochondrial marker (mitochondrial non-glycosylated protein (MTC02, 60 kDa) (ab3298), and anti-interleukin-6 (IL6 50 kDa) (ab9324). In addition, we used primary antibodies from Thermo Fisher Scientific: anti-lysine (K)-specific demethylase 6A (KDM6A, 140 kDa) (PA5-68598), anti-hypoxia-inducible factor 1-alpha (HIF1α, 115 kDa) (PA5-85494), anti-artrophy-1 protein (OPA1, 100 and 80 kDa) (BDB612607), anti-phospho-dynamin related protein 1 (Ser616) (Phospho-DRP1) (PA5-64821, 95 kDa), and anti-lactate dehydrogenase (LDH, 35 kDa) (PA5-27406). Cell Signaling Technology (Danvers, MA, USA) provided antibodies: anti-extracellular signal-regulated protein kinase (ERK1/2, 42/44 kDa) (#4695), anti-phospho-ERK1/2 (Thr202/Tyr204), (42/44 kDa) (#9101), Merck (Darmstadt, Germany) provided anti- dynamin related protein 1 (DRP1, 74–95 kDa) (ABT155), while the anti-hexokinase I (HK I, 120 kDa) (sc-80978) was obtained from Santa Cruz Biotechnology (Dallas, TX, US). The appropriate horseradish peroxidase-conjugated secondary antibodies were used. The expression levels of anti-glyceraldehyde-3-phosphate dehydrogenase (GAPDH, 37 kDa) (ab9485) and β actin (42 kDa) (CP01, Calbiochem), as well as Ponceau staining were used as loading controls for normalization. The blots were cut before hybridization with antibodies during blotting. Uncropped images of blots and exemplary blot images used for densitometric analysis are shown in Supplementary Figs. S2, S3, S4,  5a and 5b. Protein bands were visualized using the SuperSignal ECL substrate (Thermo Fisher Scientific) and were digitally quantified using the ImageJ software package.

### Statistical analysis

Data are presented as means ± SD of 5–10 independent preparations of cell suspensions. Each measurement was performed in at least two replicates. ANOVA (followed by Tukey’s post hoc comparisons for *P* < 0.05) or nonparametric Kruscal-Wallis ANOVA (followed by Dunn's post hoc comparisons for *P* < 0.05) was used to determine statistically significant differences (*P < 0.05, **P < 0.01, ***P < 0.001).

### Supplementary Information

Supplementary Figure S1.

Supplementary Figure S2.

Supplementary Figure S3.

Supplementary Figure S4.

Supplementary Figure S5a.

Supplementary Figure S5b.

## Data Availability

All data generated or analyzed during this study are included in this published article (and its Supplementary Information files).
