# Supplementary figures and images for: The bisphosphonates alendronate and zoledronate induce adaptations of aerobic metabolism in permanent human endothelial cells
Source: Sci Rep. 2023 Sep 27;13:16205. doi: 10.1038/s41598-023-43377-3 (PMC10533870; doi:10.1038/s41598-023-43377-3)

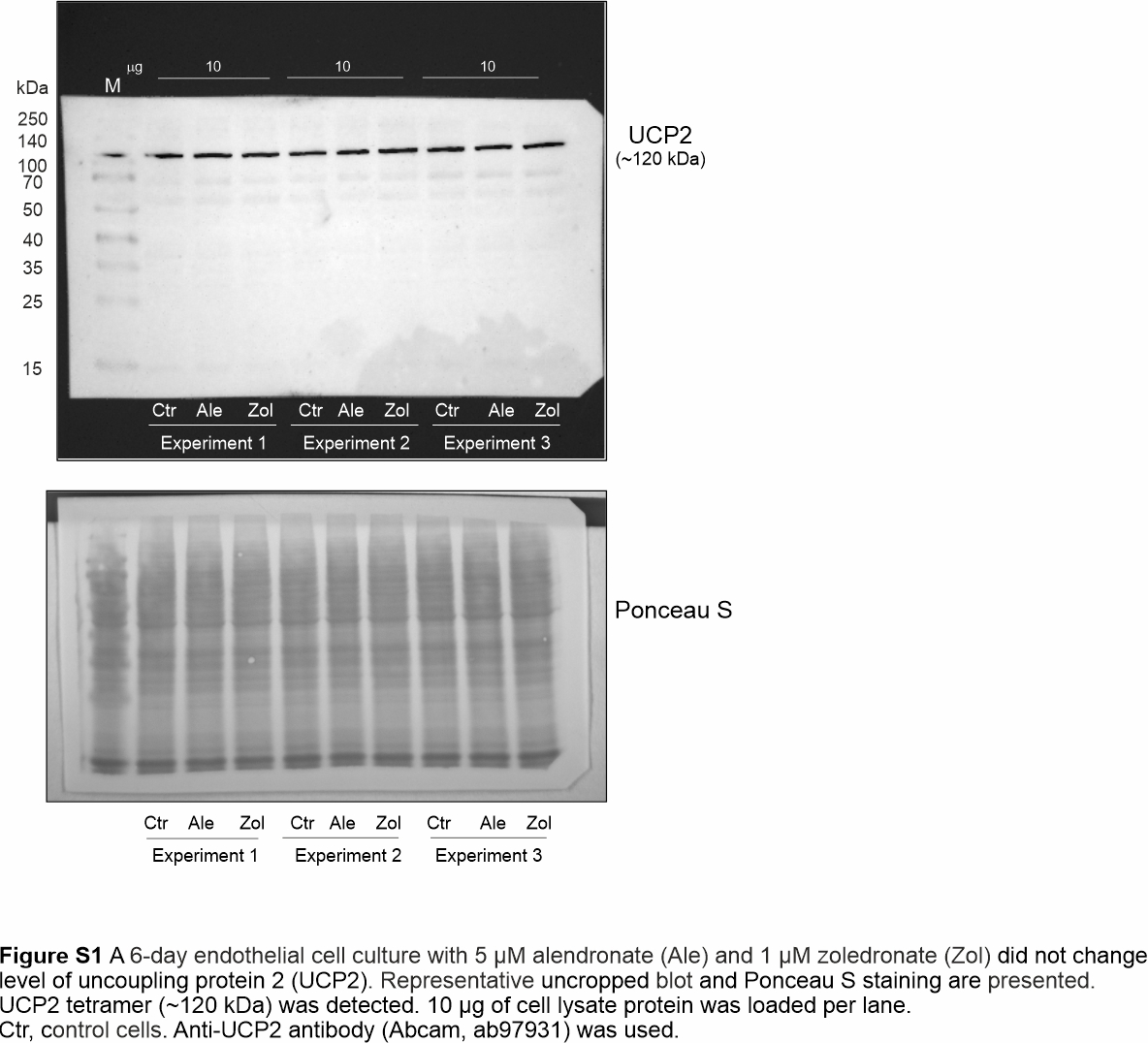

Supplement: Supplementary file 1 — Supplementary Figure S1. [file 41598_2023_43377_MOESM1_ESM.jpg]

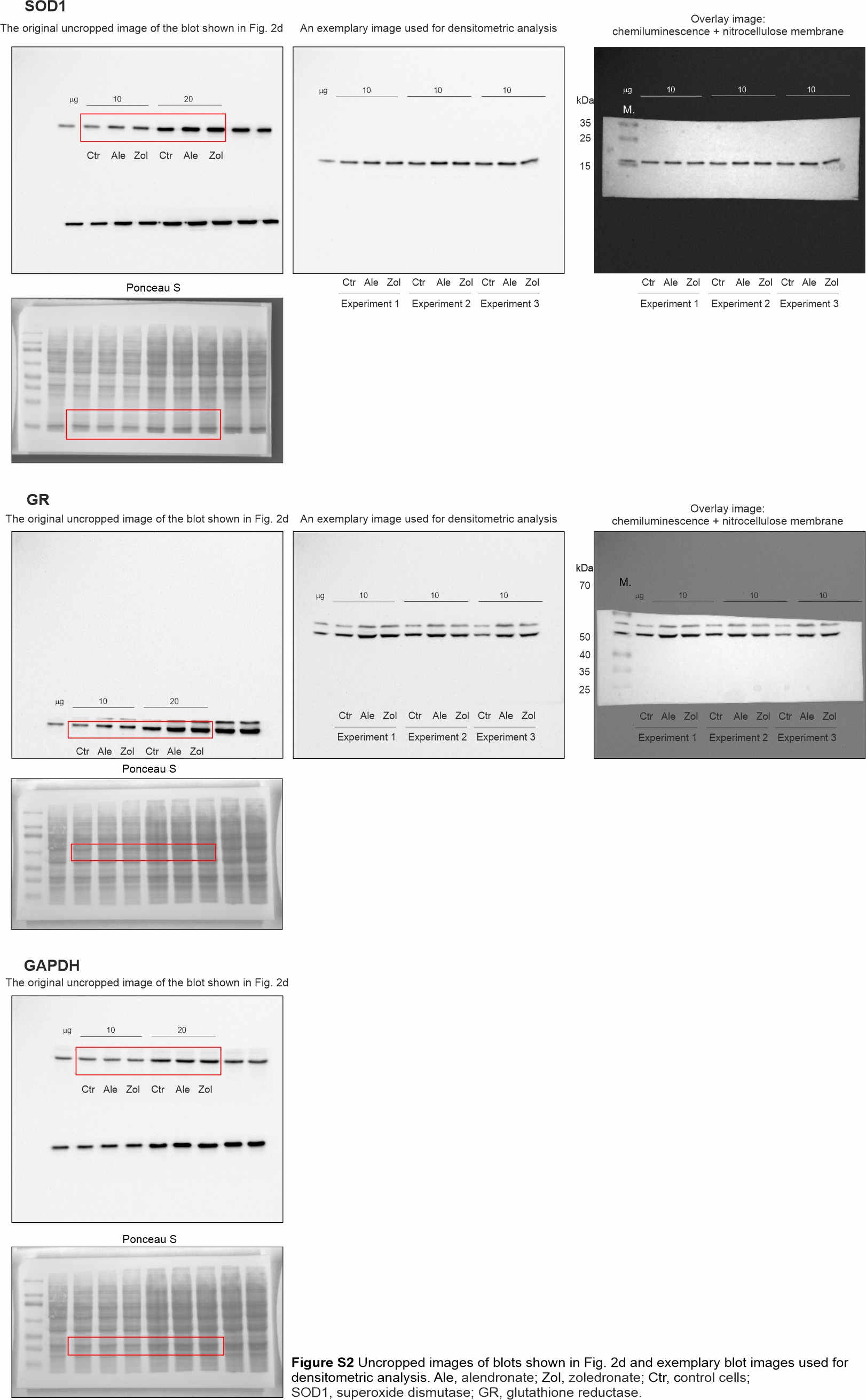

Supplement: Supplementary file 2 — Supplementary Figure S2. [file 41598_2023_43377_MOESM2_ESM.jpg]

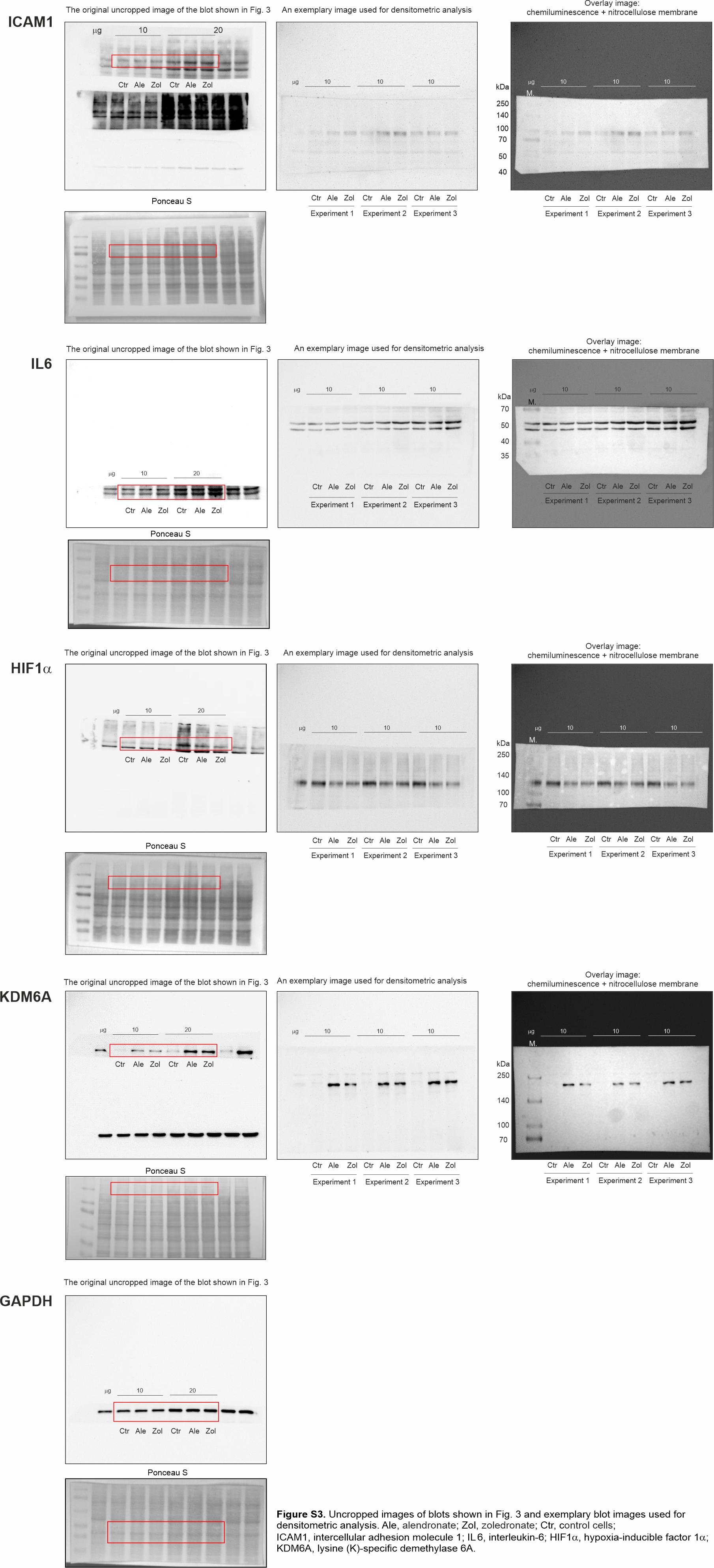

Supplement: Supplementary file 3 — Supplementary Figure S3. [file 41598_2023_43377_MOESM3_ESM.jpg]

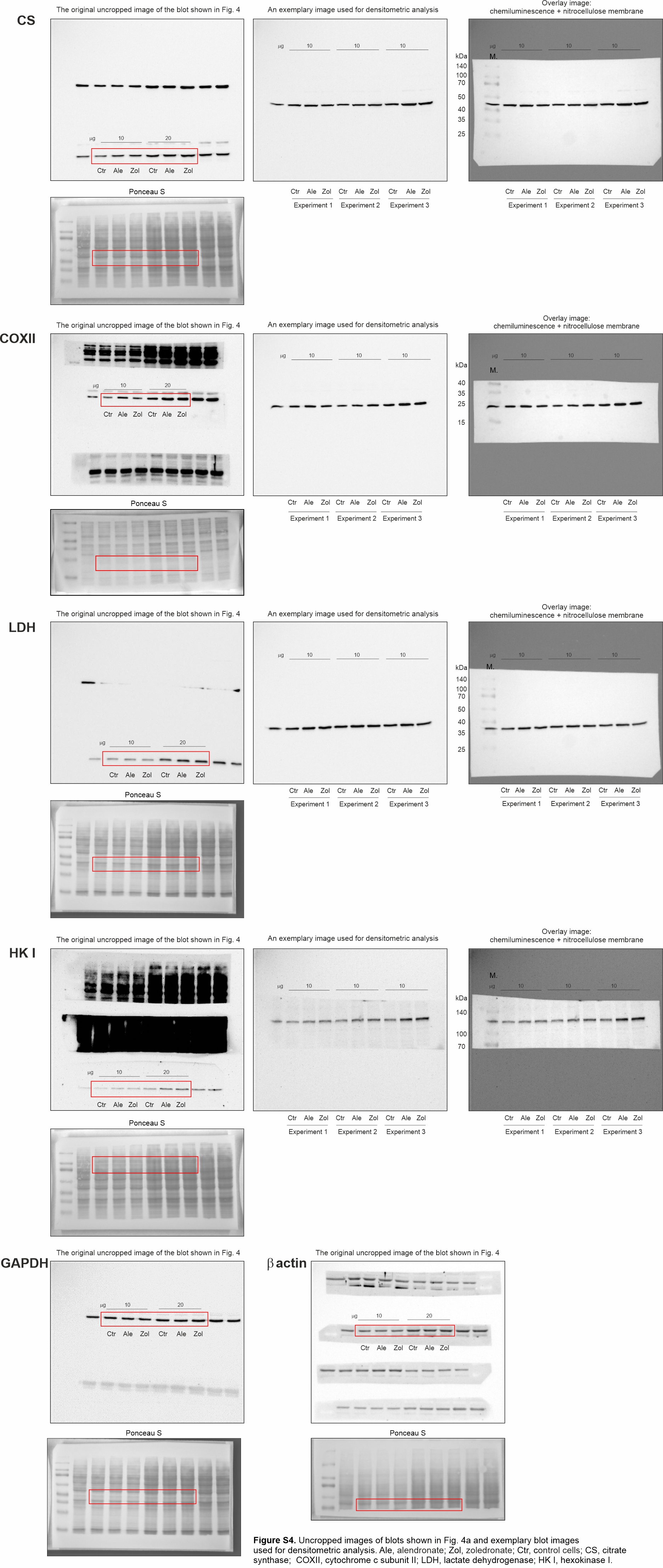

Supplement: Supplementary file 4 — Supplementary Figure S4. [file 41598_2023_43377_MOESM4_ESM.jpg]

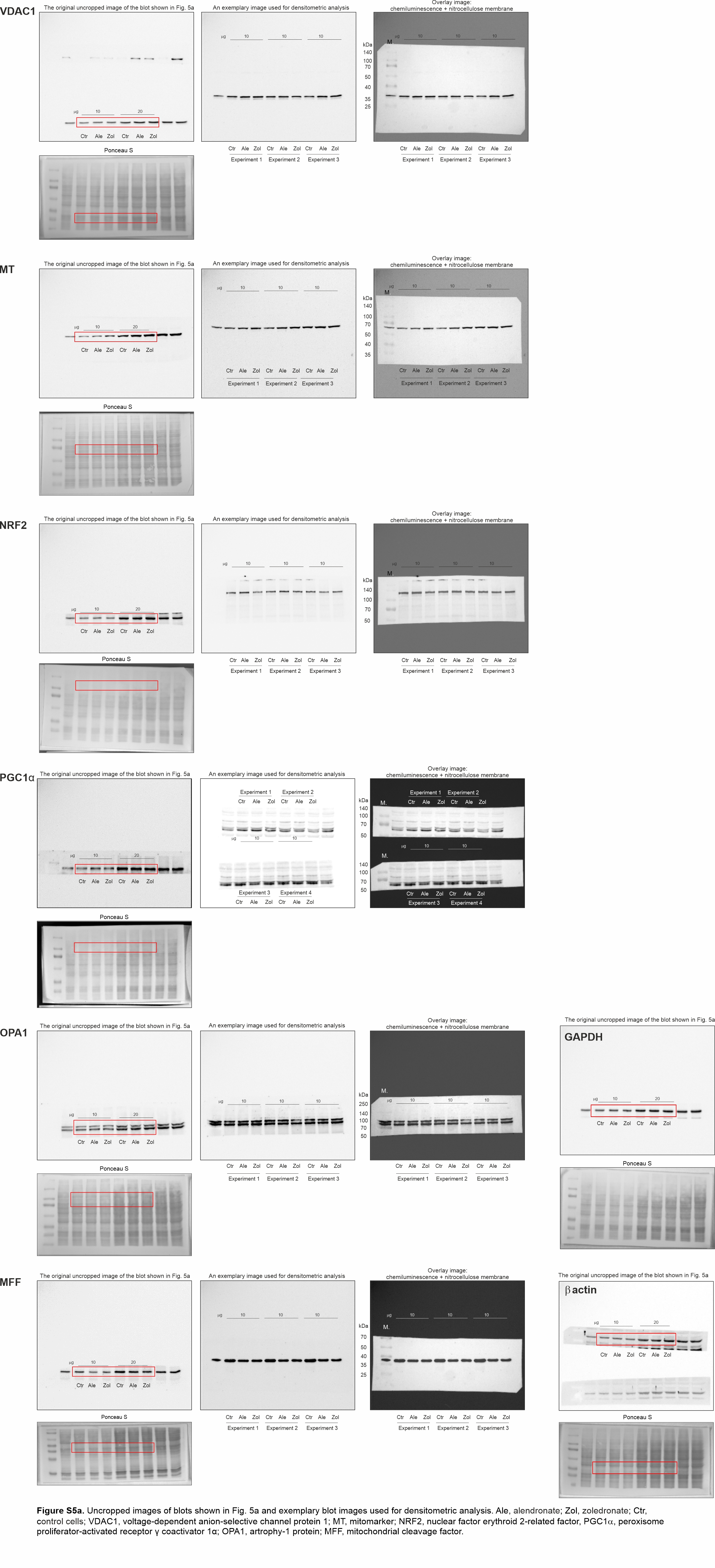

Supplement: Supplementary file 5 — Supplementary Figure S5a. [file 41598_2023_43377_MOESM5_ESM.jpg]

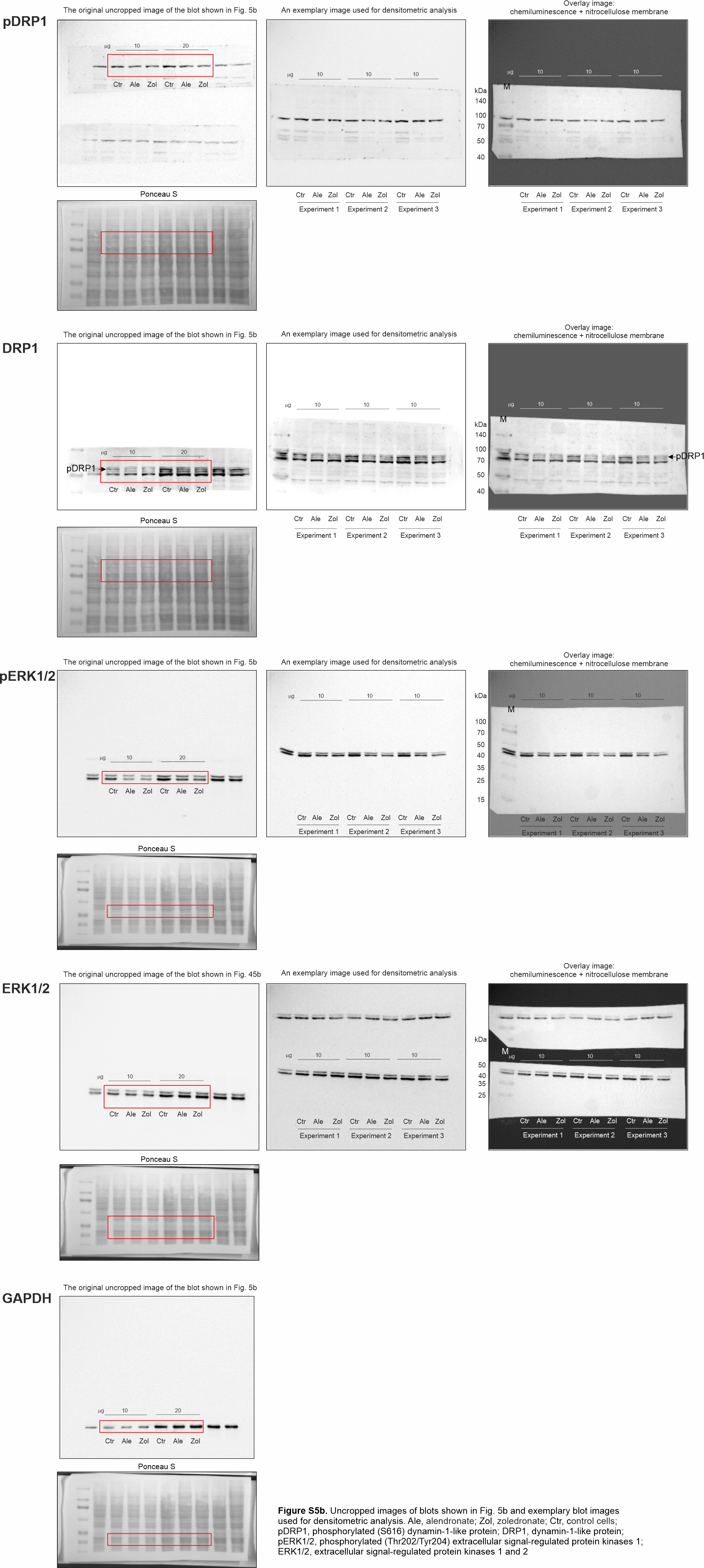

Supplement: Supplementary file 6 — Supplementary Figure S5b. [file 41598_2023_43377_MOESM6_ESM.jpg]
